# Tailoring the Properties of Biochar-Filled Composites by Pyrolysis Temperature: A Review

**DOI:** 10.3390/polym18111318

**Published:** 2026-05-27

**Authors:** Giulia Infurna, Nadka Tz. Dintcheva

**Affiliations:** Dipartimento di Ingegneria, Università degli Studi di Palermo, Edificio 6, 90128 Palermo, Italy; nadka.dintcheva@unipa.it

**Keywords:** biochar, pyrolysis temperature, sustainable filler, waste recovery, physicochemical properties

## Abstract

Biochar, a carbon-rich material derived from biomass pyrolysis, offers a promising pathway for valorising agricultural and industrial residues within a circular economy. This review analyses the evolution of biochar properties, including fixed carbon content, elemental composition, surface functional groups, porosity, pH, hydrophobicity, and thermal stability, as a function of pyrolysis temperature. The novelty of this work lies in the systematic correlation between the thermal history of biochar and its performance as a functional filler in polymer composites. In fact, increasing temperature enhances carbonisation and aromatic ordering, and in turn induces a transition from hydrophilic to hydrophobic behaviour, thereby promoting micro–mesoporous development. These shifts are critical for compatibility with polymer matrices and thus the production of light-weight, cost-effective, and environmentally friendly composite materials through processes such as melt extrusion and injection moulding. This study highlights how biochar can be tuned for compatibility: low-temperature biochar enhances adhesion in polar systems, while high-temperature biochar favours non-polar matrices, improving stiffness, thermal stability, and electrical conductivity. In biodegradable polymer composites, additional effects on crystallisation behaviour and degradation mechanisms emerge, further highlighting the complexity of designing biochar-reinforced systems.

## 1. Introduction

The effects of temperature on the physical and chemical properties of biochar play an important role in composite formulation and its properties. Thermochemical decomposition processes, which include slow, fast, and flash pyrolysis, torrefaction, and gasification, are ways of converting biomass into biochar and other phases (i.e., high-weight hydrocarbon, light-weight hydrocarbon) [1]. All these processes differ in the operating conditions used, such as temperature, and each of them favours the production of either the solid phase, named char or biochar (in the case of biomass feedstock), or the mixed liquid phase of the heaviest hydrocarbon, fuel, or mixed gaseous phase of the lightest hydrocarbons, syngas.

The temperature employed, together with the heating rate, pressure, and residence time, determines the type of plant required for biomass conversion and the preferred products of the thermal decomposition section (see Table 1). Slow pyrolysis is characterised by slow heating rates and long residence times at atmospheric pressure, with operating temperatures varying from 350 to 800 °C; the energy required for the pyrolysis of the feed is usually provided internally by combustion of a portion of the feed. The main product is a high-carbon solid char, and the co-products are an aqueous, low-molecular-weight liquid and a low-energy combustible gas [2,3,4]. Fast pyrolysis is carried out at temperatures between 400 and 600 °C. In contrast to slow pyrolysis, it uses a very high heating rate in a vacuum atmosphere, a short residence time, and rapid quenching of the vapour, as the main aim of this process is to produce bio-oil [2,5,6]. Flash pyrolysis is a batch process with an operating temperature range between 300 and 800 °C and is similar to slow pyrolysis but with a high heating rate, using moderate pressure (between 2 and 25 atm) to condense volatile elements and promote secondary formation since the aim of this process is to produce a liquid biocarbon fraction or a solid phase of biochar [7]. Torrefaction is a slow pyrolysis process with a lower temperature range, between 200 and 300 °C, which mainly removes water and some volatile compounds from the biomass to produce a “brown” char that is easy to grind. This is a stabilised and friable biomass [8]. Gasification is characterised by a high process temperature (between 750 and 1800 °C) with limited and controlled oxygen concentration (usually calculated relative to stoichiometric combustion) [9] and/or steam [10]. As the name suggests, the primary products are called syngas, a non-condensable gas mixture that is essentially composed of the presence of CO and H_2_, with a smaller amount of carbon dioxide, methane, and other low-molecular-weight hydrocarbons [11].

### Biochar Yield as Function of Pyrolysis Temperature

The yield of products such as biochar is primarily determined by the influence of the operating conditions and the temperature used in the thermal degradation process. For the same type of feedstock, the yield of biochar decreases as the pyrolysis temperature increases. The results reported by Chen et al. [12] of the slow pyrolysis of bamboo carried out at different temperatures (from 300 to 700 °C) showed how the biochar yield decreases from 49.3% when the pyrolysis is carried out at 300 °C to 23.6% when it is carried out at 700 °C. Instead, the co-products increase the yield of bio-oil from 26.5% to 34.1% and the yield of non-condensable gases from 23.2% to 42.3%. For corncob biochar obtained at different pyrolysis temperatures, ranging from 300 to 600 °C, the biochar yield decreases from 77.3% to 21.7% [13]. The yield of BCs was almost reduced from 37% to 22% for soybean and peanut shell-derived biochar obtained by pyrolysis at 300 and 700 °C [14]. Similarly, when pyrolysis temperatures were increased, biochar yields decreased for several types of feedstock: hickory and hamlin citrus [15,16,17], sewage sludge [18], the residual wastes after palm oi extraction [19], residual shells of carob fruit after sugar extraction [20], woody fractions of mallee eucalyptus loxophleba [21], wheat straw and oat straw [22], pineapple peel [23], Douglas fir wood, Douglas fir bark, and hybrid poplar wood [24]. For lignocellulosic feedstocks, the low pyrolysis temperature determines the minimal condensation of aliphatic compounds and lower losses of CH_4_, H_2_, and CO. Above a certain temperature, which depends on the nature of the lignocellulosic feedstock, the yield decreases significantly due to the dehydration of hydroxyl groups and thermal degradation of lignocellulosic structures [25,26]. For the same reasons—also in the fast pyrolysis regime, which implies a higher heating rate—a study of the effect of operating conditions, such as temperature, on pine wood-derived biochar was carried out. In terms of biochar yield as a function of temperature, the trend remains unchanged; in fact, a decrease from about 42 wt.% of dry biomass (obtained by fast pyrolysis at 325 °C) to about 11 wt.% dry biomass (obtained by fast pyrolysis at 575 °C) was shown, with a decreasing curve that shows an exponential decrease [27].

## 2. Biochar Properties as a Function of Pyrolysis Temperature

### 2.1. Fixed Carbon

Proximate analysis is a useful analysis carried out on feedstock to determine the suitability of the feedstock for conversion to biogas, biofuel, or biochar, as it provides information on the fixed carbon, volatile matters, and ash in the feedstock [28]. The fixed carbon represents the solid carbon in the biomass that is expected to remain in the char after devolatilisation in the pyrolysis process [29].

At the same time, proximate analysis could be a great resource for characterising the biochar after the pyrolysis process, as the fixed carbon of the char represents the material that remains after the volatiles have been driven off [30], and this data is certainly highly dependent on the process temperature. Proximate analysis can be used also to understand the number of residual volatiles in biochar samples, which are the product of insufficient thermal degradation during the pyrolysis process. Thus, as the process temperature increases, the amount of volatile matter decreases and the amount of fixed car-bon increases. Moreover, due to the accumulation of mineral components resulting from the decomposition of organic constituents, the ash content of biochar increased with increasing production temperature. Several studies confirm these trends in fixed carbon, volatile matter, and ash contents as a function of pyrolysis temperature. Chen et al. [12] pyrolysed bamboo from 300 to 700 °C and performed a proximate analysis on the resulting biochar, showing that volatile matter decreases as the temperature increases, from 30% at 300 °C to 9% at 700 °C, and the relative fixed carbon increases from 65% at 300 °C to 86% at 700 °C. The same trend has been reported by proximate analysis of the resulting biochar derived from the pyrolysis of soybean stover and peanut shell [14], corn cob [13], and cocoa pod waste coming from the cocoa industry, as well as the resulting digestate of cocoa pods for biogas production [31], coconut fibre and pine wood [15], and wood hybrid poplar (*Populous deltoids*) and Douglas fir *(Pseudotsuga menziessii*) wood and bark [24].

### 2.2. Elemental Composition

An important parameter of biochar is its elemental composition; total carbon, nitrogen, and hydrogen in biochar samples and, in some studies, sulphur, are often analysed with a CHN elemental analyser, and oxygen content is often obtained as the weight difference between raw biochar and the sum of C, H, N, and non-volatile elements. C, H, N, and O are elements that are highly sensitive to pyrolysis temperature, unlike non-volatile and inorganic elements, whose total content depends more on the nature of the pyrolysed feedstock. In particular, as the pyrolysis temperature increases, the biochar becomes richer in carbon, while the oxygen and hydrogen content decreases. The ratios of hydrogen to carbon (H/C) and oxygen to carbon (O/C), which are considered pyrolysis indicators, can be calculated from these quantities, and there is a correlation between these two ratios and the chemical structure of the resulting biochar. In fact, as hydrogen or oxygen decreases relative to the feedstock and relative to the total carbon content, the formation of aromatic structures occurs, as well as the condensation of carbon atoms through the loss of O-containing functional groups. As mentioned earlier, if biochar becomes richer in carbon while the oxygen and hydrogen content decreased with increasing temperature, it follows that the H/C and O/C ratios decrease with increasing pyrolysis temperature. Thus, at a higher temperature, the biochar gradually becomes richer in carbon with an aromatic structure. These results are largely confirmed by the experimental study present in the literature and for several types of biomass, woody, and non-woody waste: moso bamboo [12]; corn-cob, sawdust, and cornstalk [13]; soybean stover and peanut shells [14]; loblolly pine, citrus, alfalfa, and switchgrass [17]; pine wood and coconut fibre [15]; hickory wood, bagasse, and bamboo [16]; peanut hull, pecan shell, poultry litter, and switchgrass [26]; carob waste [32]; wood apple shells [33]; cassava rhizome, durian peel, and pineapple peel [34]; wood [35]; cocoa pod waste coming from the cocoa industry and the resulting digestate of cocoa pods for biogas production [31]; palm waste [36]; pine needles [37]; and others [38,39,40,41,42,43,44,45,46,47,48,49,50,51]. The H/C and O/C ratios decrease as the pyrolysis temperature increases, regardless of the variation in heating rate and thus, in a sense, the type of pyrolysis used (slow or fast). In fact, Amine Hmid et al. [52] have shown that the pyrolysis of olive mill waste at pyrolysis temperatures of 430, 480, and 530 °C at different heating rates (i.e., 25, 35, and 45 °C/min) results in an increase in carbon content and a decrease in oxygen and hydrogen content with increasing pyrolysis temperature. Likewise, Onay fast-pyrolysed safflower seeds for biochar production at pyrolysis temperatures of 400, 600, and 700 °C at much higher heating rates (i.e., 100, 300, and 800 °C/min), confirming the same carbon, hydrogen, and oxygen content trends as previously shown [53].

### 2.3. Surface Functional Groups

**Figure 1:**
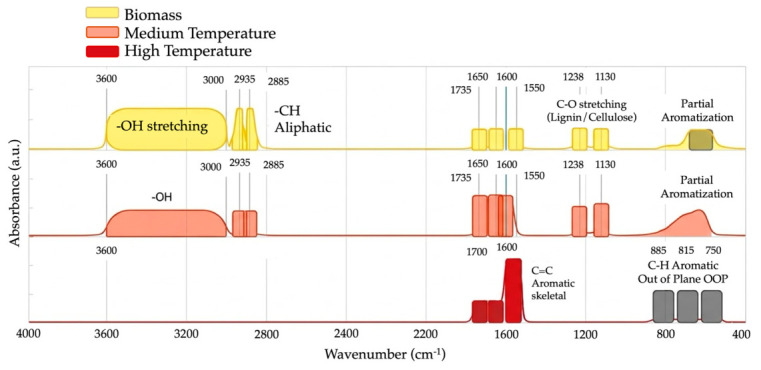
Qualitative evolution of FT−IR spectra from raw biomass to biochar produced at different pyrolysis temperatures.

Considering how the variation in elemental composition contributes to the variation in the biochar chemical structure as pyrolysis temperature varies, complex molecular chains (such as cellulose, lignin, and hemicellulose) are transformed into very different chemical structures, with the appearance or disappearance of functional groups on the biochar surface, strictly dependent on pyrolysis temperature. Figure 1 qualitatively illustrates the variation in FTIR peak intensities from biomass to biochar as a function of pyrolysis temperature. Indeed, the FTIR spectra of biochar are dominated by functional groups typical of oxygenated hydrocarbons, reflecting the carbohydrate structure of cellulose and hemicelluloses. Pyrolysis can cause absorption bands characteristic of raw materials to disappear and new bands typical of biochar samples to appear.

A typical biochar FT−IR spectra (schematised in Figure 1) is normally composed of a broad band from 3000 to 3600 cm^−1^ attributed to the presence of OH functional groups (alcoholic and phenolic) [54,55]; a smaller band from 2700 to 3000 cm^−1^ attributed to alkyl C–H stretching [56]; a band at 1600 cm^−1^ attributed to the aromatic C–C and C–O stretching of conjugated ketones and quinones [57]; a band occurring at 1735 cm^−1^ attributed to the C=O stretching of ketones, aldehydes, and esters [58,59]; a band centred at 1238 cm^−1^ attributed to the presence of C–O–C groups and aryl ethers; phenolics associated with lignin [60]; and a band at 1130 cm^−1^ attributed to the C–O–C stretching of ester groups in cellulose and hemicelluloses [56]. When heated to a high temperature, the chemical bonds are broken and rearranged and new functional groups are formed, in which it is possible to distinguish them as acidic (i.e., carboxyl, carbonyl, lactone, phenol, pyrone, ether, anhydride) and basic groups (i.e., pyridine, pyrrole) [61]. Suliman et al. [24] showed how the increase of the pyrolysis temperature of three different biomasses determines a linear decrease in the content of oxygenated functional groups (i.e., phenolic, lactone, and carboxylic groups) and a reverse trend for the total basic surface functional groups. All these functional groups are present on the outer surface of biochar and in its pores [62,63,64,65]. Biochar produced at low temperature displays more of these functional groups due to the retainment of an aliphatic and cellulose structure [26,56], and a higher temperature leads to dehydration and deoxidation of the biomass with a consequent highly hydrophobic nature with well-organised C layers [58,66]. In particular, the band related to the -OH stretching due to hydrogen-bonded hydroxyl groups (3200–3500 cm^−1^) is the first to disappear with increasing pyrolysis temperature for biomass-derived biochar. Then, the asymmetric (2935 cm^−1^) and symmetric (2885 cm^−1^) C−H stretching of aliphatic functional groups decrease and then disappear, as does the symmetric C–O stretching (1030 and 1110 cm^−1^). Instead, for the carboxylic acid C=O (1700–1740 cm^−1^), the aromatic C=C stretching of conjugated ketones and quinones (1600 cm^−1^) first increases and then decreases with the increase of the pyrolysis temperature. As the temperature increases, the C−H bending for aromatic out-of-plane deformation (750, 815, and 885 cm^−1^) becomes more prominent, reaching a spectrum at the highest temperature that could be related to that of pure graphite [35,51,60,67,68,69].

### 2.4. Surface Area and Porosity

**Figure 2:**
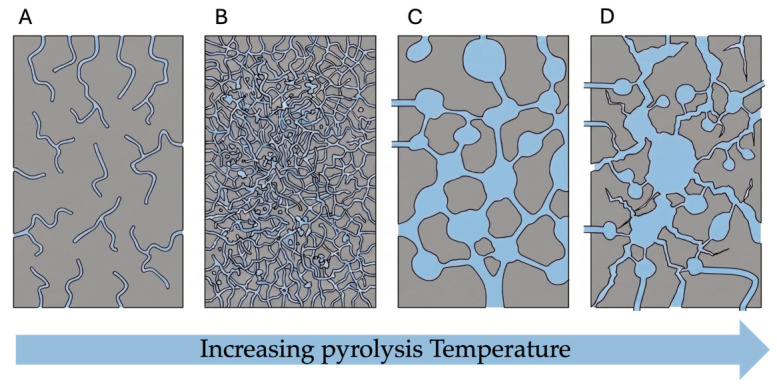
Schematic evolution of pore morphology as a function of pyrolysis temperature on biochar formation. (**A**) Development of microporosity and isolated channels; (**B**) formation of an interconnected pore network; (**C**) transition to macroporosity via pore coalescence; (**D**) emergence of structural fractures and cracks.

The surface area of the biochar is one of the key characteristics that can greatly influence its behaviour, helping to absorb chemical compounds, affecting its reactivity and the combustion characteristics of the biochar. It is a parameter that depends on the nature of the feedstock since a complex lignocellulosic structure already has a high surface area with a relatively high pore volume, as well as the temperature used in the pyrolysis process. The devolatilisation step promoting pyrolysis process seems to be responsible for the formation of pores [17]. By increasing the driving force of the devolatilisation process, the increase in pyrolysis temperature changes the morphology of the char particles (see Figure 2). The higher temperature causes a rapid release of volatiles during pyrolysis. This leads to an internal overpressure, which causes smaller pores to merge. This merging process results in the formation of large internal voids with a porous biochar structure; hence, the macroporosity increases with increasing temperature [13,16,51,66,68]. A higher pyrolysis temperature also creates a porous structure due to several cracks and holes generated by the releasing of volatile matter [70]. Sizirici et al. [36] showed a BET surface area that increased from 1.1 m^2^ g^−1^ and 3.5 m^2^ g^−1^ of the frond and leaf, respectively, of palm waste pyrolysed at 400 °C to 246 m^2^ g^−1^ and 289 m^2^ g^−1^ when pyrolysing them at 600 °C. The orange peel-derived biochar showeed a surface area of 22.8 when pyrolysed at 150 °C, and 201 when pyrolysed at 700 °C [41]. A complete characterisation of pore structure and surface area performed by Tsai et al. [23] correlated the pore structure on adsorption–desorption isotherms, showing the coexistence of microporous and mesoporous biochar structures, and their value increases with the increase of pyrolysis temperature and residence time. In contrast, fast pyrolysis with higher heating rates results in a faster devolatilisation step that affects the resulting morphology of the biochar. Onay et al. [53] showed that pyrolysing at a heating rate of 100, 300, and 800 °C/min at three different temperatures, i.e., 400, 600, and 800 °C, yielded low values of surface area at every heating rate and pyrolysis temperature, and for all heating rates, the surface area first slightly increases then decreases with the increase of pyrolysis temperature. The decrease in surface area value observed at high temperature is due to structural ordering and the decrease in the number of micropores and increase in macropores due to the evolution of volatile bubbles at higher devolatilisation rates.

### 2.5. High Heating Value

Another important parameter affected by the pyrolysis temperature is the high heating value (HHV), which is defined as the energy released per unit mass or volume of fuel when the fuel is completely burnt. In the case of solid fuels, such as biochar, this is referred to as unit mass. There are two methods of assessing HHV: the first is experimental and is carried out using bomb calorimeters, and the second is theoretical and uses elemental composition for the calculation. Since elemental composition is known to be affected by pyrolysis temperature, this property is again dependent on pyrolysis temperature. Generally, the theoretical methods employed for HHV calculation are based on Dulong’ s formula [71], and its modification after experimental data fitting [42,72] is summarised in the following equation:

$$HHV=aC_{wt.\%}+bH_{wt.\%}+cO_{wt.\%}+{dS}_{wt.\%}+{eN}_{wt.\%}+{fA}_{wt.\%}$$

C, H, O, N, S, and A represent carbon, hydrogen, oxygen, nitrogen, sulphur, and ash, respectively, and the coefficients depend on the nature of the fuel. From an experimental point of view, Azargohar et al. [38] found that for biochar produced from wheat straw and flax straw, the heating value increased with an increase in the pyrolysis temperature, while for biochar derived from saw dust and poultry litter, no trends were found. This may be due to a decrease in the oxygen content because of the pyrolysis temperature for the first two raw materials, which is different from the others. Again, the highest value of HHV correlated to the lower oxygen content and lower H/C ratio when canola meal was pyrolysed at different temperatures [39]. The HHVs of biochar prepared at 300, 400, 500, 600, and 700 °C were 25.9, 29.8, 27.4, 26.1, and 27.2 MJ/kg, respectively. This data shows that the biochar produced at a pyrolysis temperature of 400 °C had the largest HHV, and that the maximum difference between HHVs was 15%. The larger HHVs of biochar relative to the parent biomass (20.1 MJ/kg) were expected on the basis of the lower oxygen content and H/C ratio for the biochar relative to canola meal. The energy recovery of biochar is defined as the percent energy content (yield × HHV) of biochar relative to that for a unit mass of canola meal. The energy recovery of biochar decreased from 64.9 to 43.0% with increasing temperature from 300 to 700 °C. The decrease in the energy recovery of biochar with pyrolysis temperature is due to the decrease in biochar yield with increasing pyrolysis temperature and the narrow range of biochar HHVs (25.9−29.8 MJ/kg). Other studies demonstrate how, as the pyrolysis temperature increases, the decrease of H and O content in synergy with the increase of carbon content increases the HHV values [12,15,20,42,52]. However, it has been reported that the ash content increases as the pyrolysis temperature increases. The contribution of ash to the HHV is contradictory to the increase in carbon content. In fact, as the ash contribution increases, the HHV decreases. In one study, the ash content results are predominant, strongly decreasing the HHV value [18]. In another study, as the pyrolysis temperature is increased, first, the contribution of carbon is predominant, and thus the HHV value increases, and then the amount of ash determines the decrease of HHV [40].

### 2.6. pH

The pH of biochar depends first on the pH of the biomass feedstock. However, biochar is generally alkaline or has a higher pH than the starting biomass. The alkaline nature of biochar can be attributed to two factors: (i) the acidic functional groups, such as carboxyl, hydroxyl, or formyl groups, in the biomass are stripped off during pyrolysis, and (ii) the pyrolysis process leads to an increase in the concentration of metals (present as ash). Liu et al. [13] pyrolysed corncob at 300, 400, 500, and 600 °C, finding that the corncob pH results were slightly acidic and equal to 6.4 but already pyrolysing at 300 °C pH results in a pH equal to 8.1 and, at a high temperature, yields a pH equal to 10.4. Organic functional groups and inorganic alkali influenced the alkalinity of biochar. The contribution of inorganic alkali such as Na and K became significant at temperatures above 500 °C, whereas the contribution of organic functional groups decreased with increasing pyrolysis temperature as thermal decomposition progressed [73]. As noted above, as the pyrolysis temperature increases, the functional group concentration of the biochar surface decreases and the ash content increases. It follows that pH increases with pyrolysis temperature, tending to be more alkaline, and this result is well documented in the literature [14,16,24,26,38,40,74].

### 2.7. Electrical Conductivity

The electrical conductivity of biochar generally increases with pyrolysis temperature. This relationship has been observed across different feedstock types. Research shows that as pyrolysis temperature rises from lower temperatures (around 300 °C) to higher temperatures (up to 700 °C or more), biochar electrical conductivity tends to increase [75]. This pattern holds true for various biomass sources, including crop residues like rice straw, wheat straw, corn stover, and cotton stalk [75]. The relationship between temperature and conductivity appears to be consistent across different feedstock materials. Studies on empty fruit bunch and rice husk biochar confirmed that electrical conductivity increased with higher pyrolysis temperatures (350 °C, 500 °C, and 650 °C) [56]. Poultry manure biochar also showed increased electrical conductivity as pyrolysis temperature rose from 200 °C to 500 °C [76].

However, it is worth noting that wheat straw biochar showed a decrease in electrical conductivity with increasing temperature [77], suggesting that the specific feedstock type can influence this relationship. Additionally, biochar produced at very high temperatures (400–500 °C or higher) may have such high electrical conductivity that they could be unsuitable for salt-sensitive crops [76]. In fact, EC is an electrochemical property that reflects the degree of salinity in biochar. The EC of the biochar first increased with the carbonisation temperature and then tended to stabilise once the temperature exceeded a certain threshold. This behaviour can be explained by the fact that the increase in EC is due to the loss of volatile compounds, resulting in an increased concentration of mineral elements [40]. More specifically, the correlation found is not between electrical conductivity and ash content but between the concentration of potassium and sodium in the ash and electrical conductivity.

### 2.8. Hydrophobicity

The hydrophobicity of biochar is influenced by the temperature at which pyrolysis occurs, primarily because thermal conditions govern the evolution of surface chemistry and the presence of organic compounds that interact with water. At lower pyrolysis temperatures (≤400 °C), biochar frequently exhibits significant hydrophobicity due to the retention of tarry products rich in aliphatic and non-polar aromatic compounds, which coat the surface and impede water uptake. These organic residues are gradually volatilised or decomposed as temperature increases, leading to a reduced hydrophobic character at intermediate to high temperatures (≥500–600 °C) and a shift towards surfaces richer in carbonised aromatic structures with fewer polar functional groups available for water interactions. This transition is reflected in measurements such as molar H/C and O/C ratios and contact angles, all of which tend to indicate weaker water repellence at higher temperatures as surface polarity changes and volatiles are lost. Additionally, although higher temperatures enhance aromaticity and structural stability, they also create larger pore networks that can facilitate water penetration, further diminishing surface hydrophobicity [78,79,80,81]. Marshell et al. documented that biochar produced at lower pyrolysis temperatures (≤400 °C) was strongly hydrophobic because of retained aliphatic and aromatic tarry residues coating the surface, but hydrophobicity was markedly reduced or eliminated in samples pyrolysed at and above 500–600 °C, as these volatile coatings were removed and polar surface functionalities were diminished [78]. Supporting this, Kameyama et al. demonstrated via molar ethanol droplet (MED) indices that biochar formed at 400 °C was strongly to extremely hydrophobic, whereas biochar produced at 600 °C and 800 °C exhibited much weaker hydrophobicity or even hydrophilic behaviour, consistent with surface chemistry shifts (lower H/C and O/C ratios) induced by higher temperature pyrolysis [79]. Although elevated pyrolysis temperatures have been shown to decrease long-term hydrophobicity (i.e., water repellence) by removing aliphatic tars and polar functional groups, the precise trajectory of wettability as a function of temperature can be contingent on the feedstock composition and the specific surface functional evolution.

Thus, depending on the nature of the pyrolysed feedstock and the carbonisation process that occurs following pyrolysis, it may also happen that as the pyrolysis temperature increases, hydrophobicity may also increase. Fan et al. [82] observed that at lower pyrolysis temperatures (<300 °C), the abundant -OH groups rendered the biochar highly hydrophilic. As the temperature increased to 400 °C, a significant decrease in -OH and an increase in hydrophobic groups such as =C–H and C=C were observed, resulting in the biochar becoming more hydrophobic. Subsequent increases in temperature above 400 °C did not result in substantial alterations to the hydrophobicity, despite the ongoing decrease in oxygen content. This finding suggests that the -OH groups, rather than the overall oxygen content, are the primary determinants of biochar polarity.

### 2.9. Thermal Stability

The pyrolysis temperature is the dominant processing parameter to biochar stability. The thermal stability of biochar generally increases with higher pyrolysis temperatures, reflecting progressive carbonisation, enhanced aromatic condensation, and the reduction of labile functional groups that are susceptible to thermal decomposition. As pyrolysis temperature rises, the volatile matter content decreases and the proportion of fixed carbon and fused aromatic ring structures increases, leading to chars that resist oxidation and weight loss at elevated temperatures in thermogravimetric analysis (TGA) [83]. Increasing the pyrolysis temperature causes a decrease in the content of macromolecules; these include hemicellulose, cellulose, lignin, protein, and polysaccharides. This decrease occurs in the solid residue; meanwhile, isolated aromatic rings begin to form [46,84]. In a specific study on bamboo biochar, increasing the pyrolysis temperature from 300 °C to 700 °C significantly reduced the H/C and O/C ratios while decreasing the proportion of oxidizable carbon during chemical oxidation tests, indicating the enhanced stability of the carbon structure at higher temperatures [85]. Similarly, Zhang et al. [86] demonstrated in straw-derived biochar that indicators of abiotic stability (such as R50 and oxidation-resistant carbon fraction) increase exponentially with pyrolysis temperature, with stability metrics trending toward steadily high values above ~500 °C. These changes are attributed to the thermal elimination of labile aliphatic and oxygenated groups and the increased formation of condensed aromatic microstructures that are less prone to decomposition during subsequent thermal or environmental exposure.

### 2.10. Crystalline Structure

**Figure 3:**
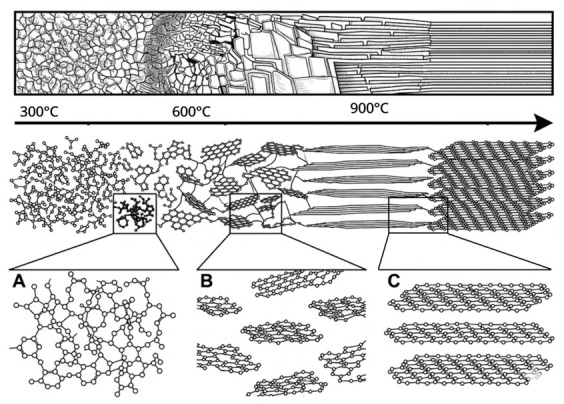
Pyrolysis temperature effect on biochar structure: A, amorphous carbon; B, turbostratic carbon; C, graphite carbon. Image adapted from Tomczyk et al. [87], licensed under CC BY 4.0.

The crystalline structure of biochar evolves significantly with increasing pyrolysis temperature, reflecting progressive transformations in the organisation of carbon atoms. At relatively low temperatures (300–400 °C), biochar typically exhibits a predominantly amorphous structure. In this range, the material retains remnants of the original lignocellulosic biomass, including disordered aromatic clusters interspersed with aliphatic chains and residual functional groups [87]. As pyrolysis temperature increases to intermediate levels (400–600 °C), thermal decomposition intensifies, leading to the removal of volatile components (removing of H and O content) and the gradual condensation of aromatic structures. This stage is characterised by the development of turbostratic carbon domains, regions where graphene-like sheets begin to form but remain randomly oriented and imperfectly stacked (see Figure 3). X-ray diffraction and Raman spectroscopy analyses commonly show increased aromatic ordering, reduced interlayer spacing, and a higher degree of structural alignment with increasing pyrolysis temperature compared to low-temperature biochar [46,88,89]. In particular, during cellulose pyrolysis, the original crystalline arrangement is progressively disrupted and transformed into polyaromatic structures, promoting the formation of turbostratic and eventually more ordered graphite-like domains as the temperature increases [90]. At higher temperatures (>600 °C), the carbon matrix undergoes further structural reorganisation, resulting in enhanced crystallinity and a more graphitic character. Aromatic layers become larger and more condensed, and the stacking order improves, although true graphite formation is rarely achieved under typical biochar production conditions. The increase in structural order is accompanied by a decrease in heteroatom content (e.g., oxygen and hydrogen) and a reduction in functional groups, leading to a more stable and chemically inert material [51].

To summarise, the physicochemical properties of biochar are strongly governed by pyrolysis temperature, as shown in Table 2. However, they are also significantly affected by the chemical nature of the feedstock, which can modulate the magnitude and direction of property changes. The simultaneous increase in fixed carbon and decrease in H/C and O/C ratios reflect the progressive aromatisation of the carbon skeleton. Notably, several properties do not follow a simple temperature-dependent trend, as multiple chemical processes occur simultaneously during pyrolysis. Non-monotonic trends are shown (↑↓) such as HHV, that result in a balance of elemental composition and ash content, which is discussed in the related section. The evolution of surface chemistry is particularly telling of the structural reorganisation occurring during pyrolysis. Specifically, functional groups such as carboxylic C=O and aromatic C=C stretching exhibit a non-monotonic trend, initially increasing and then decreasing as the temperature rises. This behaviour reflects the initial formation of oxygenated and conjugated structures followed by the final reorganisation of the carbon skeleton into more stable forms.

This behaviour is often linked to the optimal carbonisation window; for instance, while initial heating increases surface area through devolatilisation, excessive temperatures can lead to pore shrinkage or the coalescence of aromatic structures, which eventually reduces microporosity. Moreover, the shift in pH and electrical conductivity is not merely a direct function of temperature but rather a consequence of the inorganic enrichment that occurs as volatile matter is driven off. Crucially, the magnitude of this effect is strictly dependent on the nature of the feedstock: the initial concentration of alkali and alkaline metals (such as Na, K) determines the extent to which the biochar becomes alkaline or conductive at high temperatures. Therefore, the pyrolysis temperature acts as a process trigger that concentrates the mineral fraction inherently present in the starting biomass, defining the final chemical interaction with the polymer matrix. These multidimensional changes confirm that biochar is a tunable filler, where the pyrolysis temperature acts as a dial to balance thermal stability, surface polarity, and porosity for specific polymer applications.

## 3. Biochar-Filled Composite Formulation

Researchers have used carbon-based fillers such as carbon fibres, carbon nanotubes, and carbon black to improve the mechanical properties, along with making the non-conductive matrix conductive [91,92,93]. Biochar can be used as a substitute for these conventional fillers, with the result that light, sustainable composites are produced that are cost-effective and improve the carbon footprint of users throughout their lifecycle.

**Figure 4:**
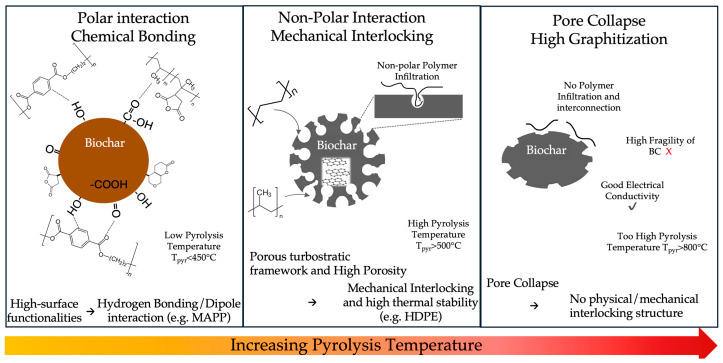
Schematic illustration of the interfacial mechanisms in biochar–polymer composites that govern the final properties of the polymer composites. (**Left**) At low T_pyr_, the biochar exhibits high surface functionality, promoting chemical bonding with polar polymers. (**Centre**) As T_pyr_ increases, biochar undergoes carbonisation and develops a porous network, enabling mechanical interlocking with non-polar polymers. (**Right**) At very high T_pyr_, the porous structure collapses, preventing effective mechanical anchoring between the filler and the polymer, leading to property reduction.

A thorough understanding of biochar’s thermal history is essential for engineering polymer-matrix compatibility and optimising downstream processing. In particular, the thermal conditions during production shape the surface functionality, porosity, aromaticity, and crystallinity that ultimately define its interaction within polymer systems. It has already been discussed that biochar produced at lower pyrolysis temperatures typically contains abundant oxygenated functional groups and disordered carbon domains, promoting stronger interfacial adhesion with polar polymers through hydrogen bonding or dipole interactions. In contrast, high-temperature biochar exhibits increased aromatic condensation, reduced polarity, and higher structural order, favouring compatibility with non-polar matrices and enhancing electrical and thermal conductivity. Thus, the thermal effects on biochar particles also extend to the final properties of the composite (as schematised in Figure 4). Increased filler crystallinity and structural ordering, typical of biochar produced at higher pyrolysis temperatures, can improve stiffness, dimensional stability, thermal resistance, and flame retardancy [94]. Nevertheless, exceeding an optimal temperature threshold can provoke a collapse of the porous structure [69], resulting in the degradation of the physicomechanical interlocking mechanisms [95]. Conversely, lower-temperature biochar, with its higher surface reactivity and defect density, may enhance impact resistance or functional performance such as adsorption or catalytic activity [96].

**Figure 5:**
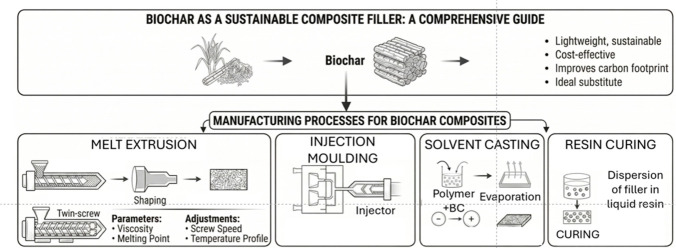
Biochar-filled composites and their main processing routes, including melt extrusion, injection moulding, solvent casting, and resin curing, together with the key processing parameters and material–process interactions governing composite fabrication.

Biochar-filled composites are primarily manufactured through standard industrial techniques (see Figure 5), such as melt extrusion and injection moulding. While these dominate the field, alternative methods like solvent casting and resin curing are occasionally employed for specific research applications [97,98].

The melt processing of polymers follows a tripartite sequence: melting, shaping, and solidification [91]. Utilising either single or twin-screw configurations, the melt extrusion relies on simultaneous heat and pressure to achieve a uniform dispersion of fillers and pigments within the polymer matrix. Efficiency is governed by intrinsic rheological properties, such as melt viscosity and melting point, and is typically optimised by adjusting screw rotation speeds and barrel temperature profiles. Injection moulding, the established paradigm for large-scale manufacturing, involves the meticulous injection of molten polymer into a precisely engineered mould cavity, thereby defining the final geometry [99,100]. As an alternative procedure, solution casting is employed to facilitate composite formation through the dissolution of polymers and additives in a suitable solvent. The process is governed by Stokes’ Law and involves blending the components into a homogeneous mixture, followed by controlled solvent evaporation to yield the final composite material [97]. In addition, epoxy-based systems are widely employed for the fabrication of biochar-reinforced composites, where the filler is dispersed in the liquid resin prior to curing. In these systems, achieving a homogeneous dispersion of biochar is a key factor influencing the final composite performance, particularly in terms of mechanical properties and interfacial adhesion [101].

Beyond these foundational techniques, a recent study has introduced specialised modifications to refine the internal architecture of biocomposites. For instance, the study demonstrated an innovative application of high-shear mixing to induce negative charges within the polymer phase, a strategy designed to engineer a segregated biochar network [102]. This approach is particularly significant as it leverages the intrinsic physicochemical properties of the matrix to customise composite performance, even if it is a methodology that remains relatively rare in the current literature. Parallel efforts to enhance interfacial compatibility have focused on surface functionalisation of the filler, and another study reported the unique multi-stage treatment of sugarcane bagasse biochar involving sequential chemical leaching with sodium hydroxide (NaOH) and hydrochloric acid (HCl), followed by thermal annealing in an isopropanol vapor environment [103]. By effectively removing surface polar groups, this protocol mitigated the inherent incompatibility between the hydrophilic biochar surface and non-polar polymer matrices, significantly improving interfacial adhesion and the overall mechanical integrity of the resulting material.

Table 3 provides an overview of the impact of pyrolysis temperature T_pyr_ on the performance of biochar-reinforced polymer composites, categorised by feedstock type, matrix, and processing conditions. The literature suggests a clear correlation between thermal treatment and the resulting interfacial dynamics, with consequent properties. At lower temperatures (e.g., 200–350 °C), biochar typically retains significant surface functionality, which enhances compatibility with polar matrices like PBAT or those utilising coupling agents like MAPE/MAPP, though it may suffer from incomplete carbonisation and lower thermal stability. Increasing toward the intermediate range (400–600 °C), there is a general improvement in the elastic modulus and tensile strength due to enhanced carbonisation and the development of porous structures that facilitate mechanical interlocking. However, the data also highlights a critical threshold: at excessively high temperatures (800–1000 °C), several studies report a pore collapse phenomenon that leads to a reduction in mechanical properties. Interestingly, at the highest extreme (°C), the biochar transitions into a functional filler capable of imparting electrical conductivity to the composite, albeit often at the expense of ductility. This summary underscores that the selection of an optimal temperature is not universal but must be precisely tuned according to the specific feedstock and the desired functionality of the final composite. A detailed discussion regarding the specific mechanical, thermal, and functional properties of these composites will follow in the subsequent sections.

Moving forward, these aspects will be further explored by categorising the composites into synthetic or biodegradable/bio-based matrices. A detailed evaluation will follow regarding the specific variations in mechanical, rheological, thermal, and functional properties as a function of the biochar characteristics.

### 3.1. Biochar-Filled Synthetic Polymer Composites

#### 3.1.1. Morphological Evolution and Mechanical Interlocking

In non-polar polyolefin matrices, the reinforcement efficiency of biochar is primarily governed by the structural transition of the filler from a disordered biomass-derived solid to a highly porous, turbostratic carbon framework as the pyrolysis temperature, T_pyr_, increases beyond 600 °C. High-temperature pyrolysis promotes the volatilisation of hemicellulose and cellulose derivatives, generating a complex micro-mesoporous architecture that facilitates mechanical interlocking through the infiltration of molten polymer chains into the filler’s pores [67,103]. This morphological evolution scales with the storage modulus, G’, and the overall tensile stiffness, as the rigid carbon skeleton acts as a superior load-bearing phase. The correlation between increased pyrolysis temperature T_pyr_ and the development of physical and mechanical interlocking between the polymer and fillers was also documented by Khan et al. [98]. In their study, the inclusion of biochar synthesised at elevated T_pyr_ into an epoxy resin provided particles with a high specific surface area. In this case, this morphological characteristic facilitated a robust interface, ultimately resulting in the transformation of the inherently brittle epoxy matrix into a more ductile composite system as a direct consequence of the filler’s high surface area.

#### 3.1.2. Mechanical Properties

Studies on polymer–biochar composites consistently demonstrate that filler production temperature governs interfacial compatibility, dispersion, and final thermomechanical performance. Arrigo et al. [107] showed that polyethylene composites filled with biochar produced at higher pyrolysis temperatures exhibited increased stiffness and thermal stability due to enhanced aromatic ordering and reduced surface polarity, which improved filler rigidity but slightly reduced elongation at break. Similarly, Bartoli et al. [108] reported that biochar structure strongly affects composite reinforcement efficiency, highlighting that highly carbonised chars act as micro-scale reinforcing phases comparable to conventional carbon fillers. The importance of feedstock and pyrolysis conditions was emphasised by Das et al. [109]*,* who demonstrated that waste-derived biochar can be engineered as a sustainable filler with tuneable modulus, electrical behaviour, and density, enabling cost-effective alternatives to carbon black and graphite in structural polymers. Expanding on this, Zhang et al. [94] demonstrated that HDPE composites reinforced with date palm-derived biochar exhibited a significant increase in elastic modulus (up to 40% with a 20% filler loading) and enhanced thermal stability. The study highlighted that the porous structure of the biochar promotes mechanical interlocking with the HDPE matrix, effectively improving creep resistance and dimensional stability, although at the expense of elongation at break. Furthermore, T_pyr_ dictates the nucleating efficiency of the filler; highly carbonised biochar serves as a potent heterogeneous nucleating agent, significantly elevating the crystallisation temperature T_c_ in HDPE and PP by reducing the critical free energy barrier for crystal formation [95,110].

Polarity differences between the matrix and the filler can lead to repulsion, resulting in poor interfacial adhesion. Additives such as compatibilisers can be included in the final composite formulation to improve interfacial adhesion. This issue arises when a non-polar polypropylene matrix is combined with biochar obtained at a medium-to-low pyrolysis temperature, which still results in a polar filler. A commonly used compatibiliser in this case is maleic anhydride grafted polypropylene (MAPP) [99,109,111] (see Figure 4). A good interfacial adhesion promotes better dispersion and no agglomerate formation, leading to improved composite properties. The literature reports that by adding 1% MAPP, a significant improvement in composite properties was observed, revealing a significant increase in tensile strength and flexural strength [111]. In the instance of biochar obtained at a high pyrolysis temperature and therefore possessing the absence of functional groups on its surface, its addition biochar to polypropylene results in no bonding between the biochar and the MAPP. Consequently, the mechanical properties do not exhibit a drastic change in the presence of MAPP [109]. In fact, a study by Ikram et al. [99] shows that the interaction between the non-polar polypropylene matrix and the non-polar biochar surface, enhanced by the high surface area of the biochar and its lack of functional groups, can still lead to an improvement in tensile strength. A study by Zhang et al. [95] investigated HDPE-based composites filled (50 wt.%) with either rice husk or biochar derived from rice husk obtained at pyrolysis temperatures ranging from 200 to 900 °C with 100 °C increments. Composites containing untreated rice husk exhibited poor mechanical performance, mainly due to the incompatibility between the polar nature of the filler and the apolar HDPE matrix. With increasing pyrolysis temperature, progressive carbonisation of the biomass led to a reduction in filler polarity, associated with the removal of oxygen-containing functional groups. This evolution improved filler–matrix compatibility and promoted mechanical interlocking, as the molten polymer penetrated the porous structure of the biochar, resulting in enhanced mechanical properties. However, a decline in tensile strength was observed for composites containing biochar produced at higher temperatures (700–900 °C). This behaviour was attributed to structural changes in the biochar [112], particularly pore deformation and collapse during melt mixing, which reduced the effectiveness of mechanical interlocking. As a consequence, the tensile properties of the composites deteriorated despite the improved chemical compatibility. Dynamic mechanical analysis (DMA) was used to study the viscoelastic behaviour of the composites. The storage modulus of all the composites was found to be higher than that of neat HDPE. The experiment revealed a decrease in the storage modulus of the composites with rising temperature. The cause of this decrease was the increase of the thermal movement of thermoplastic matrix molecules in the composite [95,112]. The highest modulus value was reported for composites filled with biochar carbonised at 600 °C due to mechanical interlocking between the matrix and filler.

A study by Bartoli et al. [101]. emphasised the central role of pyrolysis temperature in tuning epoxy-based composite performance filled with biochar. In particular, the influence of biochar production parameters (T_pyr_ and heating rate (HR)) on the mechanical performance of epoxy–biochar composites exhibited a non-linear and morphology-dependent behaviour. Biochar obtained at 400 °C with an HR of 50 °C·min^−1^ significantly enhanced the ultimate tensile strength and Young’s modulus, yielding increases of 65% and 35%, respectively. In contrast, biochar produced at the same T_pyr_ under different HR conditions did not markedly affect stiffness or strength but promoted ductility, with the maximum elongation increasing by up to 29% relative to the neat epoxy matrix. At a higher T_pyr_ (600 °C), biochar produced at 5 and 15 °C·min^−1^ induced substantial improvements in mechanical performance, with the Young’s modulus and ultimate tensile strength increasing by up to 33% and 70%, respectively. Such variations can be ascribed not only to morphology but also to T_pyr_-dependent changes in the surface chemistry. Increasing pyrolysis temperature reduces the content of oxygenated functional groups, leading to lower polarity and, consequently, weaker intrinsic affinity with the polar epoxy matrix. Notably, biochar produced at a T_pyr_ of 1000 °C resulted in the highest enhancement in ductility, with maximum elongation increasing by up to 54% compared to the unfilled system. This suggests that under in situ polymerisation conditions, the reduced polarity of highly carbonised biochar is compensated by its influence on network formation and stress transfer mechanisms. Overall, these findings highlight the critical role of the thermal history of biochar in tailoring the structure–property relationships of epoxy-based composites through the combined effect of morphology, surface chemistry, and interaction with the curing system. In this case, the evolution of the crosslinked network and the resulting interfacial interactions appear to mitigate the expected effects of polarity, pointing to a more complex, multi-factorial structure–property relationship.

#### 3.1.3. Rheological and Viscoelastic Behaviour

The rheological behaviour of the melt of non-polar polyolefins is intensified by increasing T_pyr_, causing a shear-thinning phenomenon and an increase in complex viscosity. This is attributed to the formation of a robust filler–filler percolative network and the restriction of long-range chain mobility, particularly when the biochar develops a high specific surface area at elevated temperatures [113]. Instead, from a rheological perspective, elastomer-based PP/POE composites filled with biochar exhibit a clear reinforcement effect compared to the unfilled blend, as evidenced by an overall increase in complex viscosity [100]. This viscosity increase is strongly dependent on filler loading, with higher biochar contents leading to progressively higher resistance to flow, consistent with the formation of a more constrained polymer network. However, the pyrolysis temperature of the biochar plays a crucial role in modulating this behaviour. Composites containing biochar produced at lower pyrolysis temperatures display a higher complex viscosity than those filled with high-T_pyr_ biochar at equivalent loadings. This suggests that low-T_pyr_ chars possess a more heterogeneous surface chemistry or higher content of residual functional groups, which can enhance polymer–filler interactions and promote stronger physical coupling with the PP/POE matrix [100].

Regarding the strain-dependent rheological response, systems filled with low-T_pyr_ biochar exhibit a more pronounced decrease in complex viscosity with increasing strain, indicative of stronger shear-thinning behaviour. This response can be attributed to weaker structural stability under deformation, where filler–polymer interactions are more easily disrupted and interfacial slippage occurs more readily. In contrast, composites incorporating high-temperature pyrolysis biochar show a more stable viscoelastic response under strain. These systems are likely governed by a more rigid particle network.

It has been confirmed that increasing the pyrolysis temperature generally enhances filler conductivity, hydrophobicity, and crystallinity, which promote dispersion in non-polar matrices but may reduce compatibility with polar polymers unless surface modification is applied [114].

#### 3.1.4. Electrical and Thermal Properties

Furthermore, the functionalisation of polymers through biochar addition extends to its electrical properties, transforming the insulating polymer into an electrically conductive polymer composite (ECPC). As reviewed by Das et al. [115], the electrical conductivity of these composites is highly dependent on the pyrolysis temperature and filler loading. The establishment of a percolation threshold (typically observed around 6–10 wt.% for high-temperature biochar) enables the formation of a continuous conductive network within the HDPE matrix. Biochar carbonised above 600 °C, characterised by increased graphitic carbon content, allows for the composite to reach conductivity levels (up to 10^−3^ S/cm) comparable to those achieved with technical fillers like carbon black or graphene.

Biochar carbonised at elevated temperatures (generally above 500 °C) progressively develops electrical conductivity as a consequence of increasing carbon ordering and aromatic condensation. Wood-derived monoliths subjected to pyrolysis up to 1000 °C exhibit a marked increase in fixed carbon content, which directly correlates with enhanced electrical transport. In particular, biochar produced at 1000 °C reached conductivity values in the range of 2300–3300 S/m, highlighting the strong influence of high-temperature treatment on charge mobility within the carbon matrix [116,117].

In polymer composite systems, this intrinsic conductivity becomes functional once a percolating network is established. Nan et al. [97] observed a clear insulator-to-conductor transition in biochar-filled composites prepared via solution casting, with the percolation threshold occurring at approximately 6 wt.%. At 10 wt.% filler loading, the conductivity reached 1.833 × 10^−3^ S/cm [97], a value comparable to that achieved using much lower loadings of single-walled carbon nanotubes or graphene-based fillers. However, such results also underline a key limitation of biochar systems: relatively higher filler contents are generally required to achieve similar electrical performance compared to highly engineered carbon nanofillers [118,119]. Despite this, the authors emphasise the overall advantages in terms of sustainability, low cost, and scalability, which make biochar a competitive alternative for multifunctional applications [102]. The progressive removal of oxygenated functional groups at increasing pyrolysis temperatures reduces surface polarity and modifies interfacial polarisation phenomena, thereby affecting the dielectric response and charge transport behaviour of the material. Concurrently, the increased graphitisation enhances intrinsic conductivity while also influencing interfacial compatibility with polymer matrices. Li et al. [102] demonstrated this effect in polyethylene composites filled with biochar produced at 1100 °C, where high filler loadings (60–80 wt.%) were required to achieve a well-developed conductive network, reaching a maximum conductivity of 107.6 S/m at 80 wt.%, the highest reported value for biochar-based polymer systems [120]. Similarly, coffee-waste-derived biochar carbonised at different temperatures (400–1000 °C) has been investigated as a filler in epoxy composites (5–20 wt.%). A marked increase in electrical conductivity was observed with increasing filler content and carbonisation temperature, reaching values of 2 S/m at 20 wt.% for biochar produced at the highest temperature, significantly outperforming conventional carbon black at the same loading [105].

Regarding thermal stability, the incorporation of biochar into polymer matrices generally leads to an overall improvement in thermal degradation behaviour compared to the neat polymer. Thermogravimetric analysis (TGA) studies reported in the literature consistently show an increase in thermal stability upon biochar addition, which is attributed to the intrinsically high thermal resistance of the carbonaceous filler. In particular, an increase in pyrolysis temperature results in a higher residual mass at the end of TGA experiments, in agreement with the progressively higher aromaticity and thermal stability of biochar produced at elevated temperatures. From a degradation mechanism perspective, composites containing biochar produced at relatively low temperatures (200–400 °C) exhibit derivative thermogravimetric (DTG) peaks associated with the decomposition of residual lignocellulosic components, mainly cellulose and hemicellulose, typically observed around 330 and 380 °C [95]. This behaviour indicates the incomplete carbonisation of the feedstock at lower pyrolysis temperatures. In contrast, increasing the pyrolysis temperature leads to a reduction of these devolatilisation features and a shift of the degradation onset towards higher temperatures, with thermal degradation of the composites occurring at approximately 490 °C, thus confirming an enhancement in thermal resistance compared to neat HDPE [95]. Moreover, a higher char residue is retained in composites filled with biochar produced at 600–900 °C, further supporting the role of highly carbonised fillers in improving the thermal stability of the polymer system [121]. In addition to thermal degradation behaviour, biochar also influences the thermal transitions and crystallisation behaviour of the polymer matrix. Differential scanning calorimetry (DSC) studies have shown that biochar incorporation does not significantly affect the melting temperature of polypropylene; however, it increases the energy required for melting the composites [67]. Furthermore, an increase in crystallisation temperature has been widely reported, which is generally attributed to the nucleating effect of biochar particles that promote heterogeneous crystal formation and enhance crystallisation kinetics. In some cases, variations in melting and crystallisation enthalpies have also been observed, either increasing or decreasing [103,122] depending on the specific polymer–filler system and loading conditions. Overall, the literature consistently indicates that biochar incorporation leads to an improvement in the thermal performance of polymer composites [67].

### 3.2. Biochar-Filled Biobased/Biodegradable Polymer Composites

The integration of biochar as a sustainable filler into biobased and biodegradable polymer matrices has emerged as a promising strategy to develop high-performance eco-friendly composites without compromising end-of-life biodegradability [123]. However, the final properties of such composites are not solely governed by the polymer matrix or biochar content; rather, they are critically influenced by the physicochemical properties of the biochar, which are, in turn, determined by the pyrolysis temperature and the chemical nature of the biomass feedstock [124].

Understanding this structure–property relationship as a function of pyrolysis temperature is therefore essential for the rational design of biochar-reinforced biopolymers tailored to specific applications. When shifting the focus to bio-based and biodegradable matrices (e.g., PLA, PHA, and starch), the influence of pyrolysis temperature (T_pyr_) may extend beyond mechanical reinforcement, directly affecting the hydrolytic stability and degradation kinetics of the composites [125,126]. However, the available literature remains relatively scarce in studies systematically examining a single bio-based and/or biodegradable matrix (especially in comparison with synthetic counterparts) reinforced with biochar obtained at different pyrolysis temperatures. In this section, a comparative analysis is undertaken to identify potential trends as a function of pyrolysis temperature.

As discussed in the previous sections of this work, low-temperature biochar, characterised by a high density of oxygenated functional groups (-OH, -COOH), exhibits a synergistic polarity with aliphatic polyesters, fostering strong hydrogen bonding at the interface [54,58]. However, this hydrophilic nature can accelerate hydrolytic degradation by promoting water uptake [127,128]. Conversely, high-T_pyr_ biochar, with its hydrophobic, turbostratic carbon structure, acts as a physical barrier against moisture, thereby enhancing the environmental durability of the bio-composite. Furthermore, the high surface area and porosity of highly carbonised biochar serve as potent heterogeneous nucleating agents for slow-crystallising polymers like PLA [129], significantly increasing the crystallisation rate and thermal profile.

#### 3.2.1. Rheological Properties

A key aspect that must be immediately addressed is the sensitivity of biodegradable matrices to hydrolytic degradation during melt processing [130]. A study has reported that PLA-based composites filled with biochar produced at 700 °C and subjected to melt compounding undergo degradation phenomena, resulting in a reduction in complex viscosity, along with decreases in melting and crystallisation temperatures and an overall loss of thermal stability. Similar behaviour has been observed in PBAT–PLA blends filled with biochar obtained from digestate pyrolysed at 400 °C [131], where the thermal, mechanical, and rheological properties significantly deteriorate due to interactions between the PLA and the biochar. Noteworthily, this effect becomes more pronounced with increasing PLA content, highlighting that the degradation process is primarily associated with the PLA phase and its interaction with biochar. This does not happen for PBAT matrix, which is reinforced by the addition of BC particles, resulting in an increase in complex viscosity.

**Figure 6:**
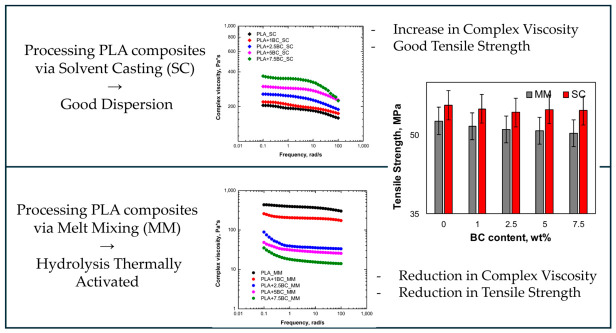
Biochar obtained at low T_pyr_ exhibits good compatibility with PLA. Biochar addition improves the properties of solvent-cast (SC) composites (**top**), while melt-mixed (MM) systems (**bottom**) undergo PLA hydrolysis, leading to a marked performance decrease. Image adapted from Arrigo et al. [127], licensed under CC BY 4.0.

Another notable study [127] compared PLA with added BC particles, processed via either solvent casting or melt compounding (schematised in Figure 6). In this study, the behaviour observed via melt compounding was confirmed by the other study, with a reduction of complex viscosity and mechanical stability, different from what was observed by processing the composites via solvent casting. However, supplementary rheological investigations have shown that even PLA-based solvent-cast materials exhibit a decrease in complex viscosity when exposed to elevated temperatures over time. In particular, monitoring the evolution of complex viscosity at 170 °C revealed that thermal exposure alone is sufficient to induce degradation, indicating that the process is thermally activated [127]. A similar reduction in complex viscosity has also been observed in polyhydroxyalkanoate (PHA) composites. In this system, the melt flow rate (MFR) decreases as the biochar content increases, meaning that higher filler loadings lead to lower MFR values and therefore reduced flowability [125].

PLA-based composites filled with biochar derived from hazelnut shell pyrolysis (300–900 °C) become progressively more fluid as the pyrolysis temperature increases [106]. This is reflected in a substantial reduction of complex viscosity due to a hydrolytic effect during processing and the enhancing of water uptake due to the presence of BC, indicating enhanced polymer chain mobility and improved filler–matrix flow alignment. At low frequencies, the materials mainly show Newtonian behaviour, while at higher frequencies, most samples exhibit shear-thinning. Increasing pyrolysis temperature also leads to a decrease in both storage and loss moduli, resulting in a more pronounced liquid-like behaviour, as confirmed by the increase in tan δ. Overall, higher pyrolysis temperatures improve the flow properties of the composites, although at the highest temperature (900 °C), the melt becomes excessively fluid, which can negatively affect filament stability for Fused Filament Fabrication (FFF) printing.

In contrast, PBAT–BC composites filled with biochar derived from carob waste (pyrolysed in a range of 280–400 °C) show a different rheological response [32]. At low frequencies, a yield-stress-like behaviour can be observed for all composites, particularly at higher BC contents. This effect becomes more evident as the biochar content increases and slightly decreases with increasing pyrolysis temperature, suggesting stronger particle network formation and more solid-like behaviour under low shear conditions.

#### 3.2.2. Mechanical Properties

In hydrolysis-sensitive polyesters such as PLA, increasing the pyrolysis temperature generally leads to a decrease in tensile strength and elongation at break, while the Young’s modulus increases. This behaviour reflects a more brittle response and premature failure of the composites. The deterioration in mechanical performance can be attributed not only to biochar (BC) aggregation, often associated with reduced particle size and higher specific surface area, which can promote stress concentration points within the polymer matrix [106], but also to degradation phenomena occurring during processing, which weakens both the polymer matrix and the BC–matrix interface [106,131]. As a result, poor interfacial adhesion and the presence of microstructural defects promote crack initiation and propagation, ultimately limiting the effectiveness of BC as a reinforcing agent, even when some improvement in fibre–matrix compatibility is achieved [106,131].

**Figure 7:**
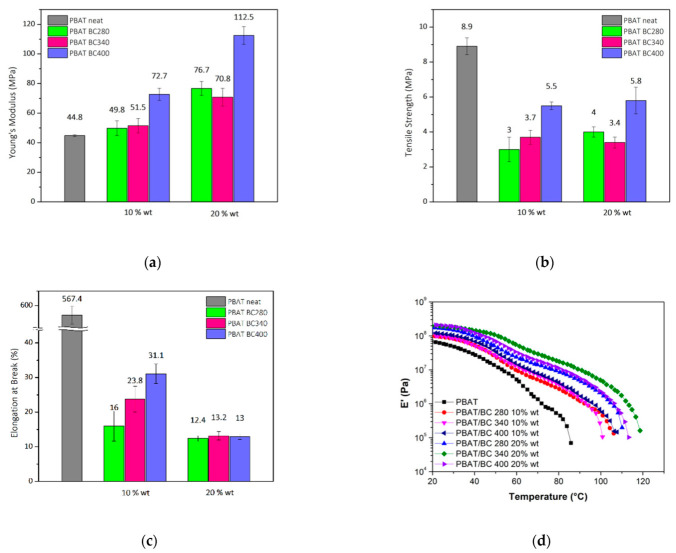
Mechanical properties of PBAT–BC composites as a function of pyrolysis temperature. Tensile properties: (**a**) Young’s modulus, (**b**) tensile strength, (**c**) elongation at break. Dynamical mechanical analysis: (**d**) storage modulus. Image adapted from Infurna et al. [32], licensed under CC BY 4.0.

Conversely, in less hydrolysis-sensitive polyesters such as PBAT, the increase in pyrolysis temperature can lead to an improvement in mechanical properties (see Figure 7). The reduction in BC particle size at higher temperatures promotes a more homogeneous dispersion, as observed by SEM analysis, and increases the interfacial area between the matrix and the filler, thus enhancing its reinforcing effect [32]. Accordingly, Young’s modulus (E) and tensile strength (TS) increase with both BC loading and pyrolysis temperature [32]. However, the addition of carbonaceous particles still induces a transition from ductile to more rigid behaviour [32]. In line with static tensile results, the storage modulus also increases with BC content and pyrolysis temperature, reflecting the same trend as the elastic modulus [32]. Nevertheless, E’ decreases with increasing temperature for all samples, although the presence of BC influences the softening behaviour of the composites.

#### 3.2.3. Crystallisation and Thermal Behaviour

In a study by Yin et al. [106], biochar particles derived from hazelnut shells were produced at different pyrolysis temperatures (T_pyr_), ranging from 300 to 900 °C, and were used as fillers for Poly-lactid-acid (PLA) for 3D-printing applications. The incorporation of hazelnut shell biochar significantly affected the crystallisation behaviour of PLA-based composites. Differential scanning calorimetry (DSC) analysis showed that biochar obtained at a high pyrolysis temperature (900 °C) enhanced the nucleating effect, thereby improving the crystallisation ability of PLA. In contrast, PLA filled with biochar produced at low T_pyr_ (300 °C) exhibited the lowest crystallinity among all composites. This behaviour can be attributed to the larger particle size at lower pyrolysis temperatures, which reduces nucleation efficiency, consistent with the known dependence of biochar particle size on T_pyr_. Furthermore, the addition of biochar promoted the thermal decomposition of the composites. This effect may be explained by the role of biochar particles as local heaters, facilitating heat transfer toward the inner regions of the PLA-based biocomposites and accelerating thermal degradation.

From a thermal standpoint, the incorporation of biochar into Poly(butylene adipate-co-terephthalate) (PBAT)-based composites slightly affects the melting behaviour but significantly influences the crystallisation process. A modest increase in the melting temperature (T_m_) is observed compared to neat PBAT, with a slight dependence on the pyrolysis temperature. In contrast, the most pronounced effect concerns the crystalline structure. The addition of biochar leads to a clear reduction in the overall degree of crystallinity, which becomes more significant as both the filler content and the pyrolysis temperature increase [32]. This indicates that biochar particles do not promote crystallisation; rather, they interfere with the regular arrangement of PBAT chains. Differently, PBAT filled with torrefied coffee grounds (obtained at low carbonisation temperature, i.e., 250–270 °C) has been reported to accelerate the crystallisation process, particularly when the filler is treated at 270 °C [104], suggesting that the thermal history and physicochemical properties of the carbonaceous filler play a key role in determining its nucleating efficiency.

The reduction in the polarity of coffee grounds induced by low-temperature thermal treatment suggests improved adhesion and dispersion within the PBAT matrix compared to untreated coffee grounds [104]. This behaviour is likely associated with the evolution of functional groups on the surface of BC, alongside the reduction in particle size observed in torrefied coffee grounds. This enhances filler embedment and promotes stronger filler–matrix interactions. Consequently, composites containing torrefied coffee grounds exhibit a more homogeneous dispersion within the PBAT matrix, although the presence of voids indicates a distribution of particle sizes.

Establishing a definitive and universal trend remains challenging due to the vast number of variables involved. The inherent heterogeneity of biomass feedstocks, coupled with the distinct interfacial chemistry of different bio-based matrices and their high sensitivity to thermomechanical degradation during processing, creates a complex landscape of interactions. This complexity, combined with a body of literature that is still relatively scarce compared to traditional synthetic polymers, precludes the formulation of an absolute structure property model. Nevertheless, the fundamental pillars of biochar evolution, specifically the shift in surface area, porosity, and surface functionality as a function of T_pyr_, provide a critical framework. Understanding these core principles serves as a strategic guide for the rational design of biocomposites, allowing researchers to tailor mechanical reinforcement, crystallisation kinetics, and environmental durability to meet the specific requirements of the final application.

The complexity of polymer–biochar interactions (arising from the decrease in biochar polarity with increasing pyrolysis temperature, the consequent variation in interactions with polar and non-polar polymer matrices, the progressive reduction of surface functional groups, and the differing chemical nature of polymers with a consequent hydrolysis sensitiveness) leads to the absence of a universal trend in composite properties. Additionally, dispersion quality and mechanical interlocking effects, as discussed in Section 3.1 and Section 3.2, further contribute to this behaviour. Table 4 summarises the observed trends by polymer class, reporting the main composite properties and their variation as a function of the increasing of pyrolysis temperature of the biochar used as filler.

## 4. Conclusions

This work highlights the central role of pyrolysis temperature in defining the physicochemical properties of biochar and, consequently, its performance as a functional filler in polymer composites. Temperature governs the transition from a reactive, oxygen-rich, and polar material to a highly carbonised, hydrophobic, and structurally ordered carbon framework. These changes affect key parameters such as surface chemistry, porosity, thermal stability, and electrical conductivity, ultimately dictating the interaction mechanisms with polymer matrices.

In composite systems, no universal optimal biochar exists; performance depends on the interplay between filler properties and matrix characteristics. Low-temperature biochar promotes chemical adhesion in polar polymers due to surface functional groups, whereas high-temperature biochar enhances mechanical interlocking, stiffness, thermal resistance, and conductivity, particularly in non-polar systems. However, excessive carbonisation may reduce interfacial compatibility or induce structural changes that limit reinforcement efficiency.

In biodegradable polymers, the behaviour is further complicated by the sensitivity of the matrix to thermomechanical and hydrolytic degradation, as well as by the competing effects of nucleation and chain mobility.

Overall, pyrolysis temperature can be used as a practical tuning parameter to design composites with targeted mechanical, thermal, and functional properties by selecting the appropriate balance between surface reactivity and the structural order of the biochar.

## Figures and Tables

**Table 1:** Main thermochemical biomass conversion processes, with corresponding temperature ranges and principal products.

Process	Temperature Range (°C)	Main Products	Refs.
Slow pyrolysis	350–800	Solid biochar; aqueous low-molecular-weight liquid; low-energy combustible gas	[2,3,4]
Fast pyrolysis	400–600	Bio-oil (primary product)	
Flash pyrolysis	300–800	Liquid biocarbon fraction and/or solid biochar	[7]
Torrefaction	200–300	Brown char; stabilised and friable biomass	[8]
Gasification	750–1800	Syngas (mainly CO and H_2_, with CO_2_, CH_4_, and light hydrocarbons)	[7,8,9]

**Table 2:** Biochar properties as a function of pyrolysis temperature.

Properties	Biochar Property Trends with Increasing Pyrolysis Temperature			Refs.
Proximate Composition	Fixed Carbon ↑	Volatile Matters ↓	Ash ↑	[12,13,14]
Elemental Content	Carbon ↑	Hydrogen ↓ $\left(\frac{H}{C}↓\right)$	Oxygen ↓ $\left(\frac{O}{C}↓\right)$	[12,13,14,15,16,17]
Functional Surface Groups *	-OH ↓	C=O ↑↓	C=C ↑↓	[35,51,57,58,67]
	CH_2 sym,asym_ ↓	C–O ↓	CH _out of plane_ ↑	
Surface Area and Porosity	Mesoporosity ↑	Microporosity ↓	Surface Area ↑	[16,66,68]
Energy Properties *	HHV ↑↓		Carbon–Ash balance	[18,40,42]
Alkalinity Properties *	pH ↑		Ash enrichment, loss of acidic group	[24,26,38,74]
Electrical Properties *	Electrical Conductivity ↑		C content and alkali metals (Na, K)	[40,75,76]
Surface Properties *	Hydrophobicity ↓			[78,79]
Thermal Properties	Thermal Stability ↑			[85,86]

* Contingent of feedstock composition; ↑ increase; ↓ decrease; ↑↓ non monotonic trend (initial increase followed by a decrease).

**Table 3:** Impact of pyrolysis temperature on the properties of biochar-reinforced polymer composites. The table summarises feedstock types, processing methods, and the resulting mechanical and thermal advantages and limitations, as reported in literature.

T_pyr_ [°C]	Matrix and Feedstock Type	Composite Processing Condition	Main Advantages	Main Disadvantage	Ref.
200	HDPE/BC from Rice Husk	Injection Moulding	-	Reduced thermal stability due to incomplete pyrolysis; lowest tensile properties.	[95]
250	PBAT/BC from Coffee Grounds	Melt mixing	Reinforcing effect.	Slight reduction in elongation at break.	[104]
270	PBAT/BC from Coffee Grounds	Melt mixing	Reinforcing effect; increased tensile strength.	Slight reduction in elongation at break.	[104]
280	PBAT/BC from Carob	Melt mixing	Improved photo-degradation resistance of the matrix.	Larger particle size; poor dispersion.	[32]
300	HDPE/BC from Rice Husk	Injection Moulding	-	Reduced thermal stability due to incomplete pyrolysis; reduction in tensile properties.	[95]
315	HDPE/BC from Wood + MAPE (3 wt.%)	Injection Moulding	Good tensile and flexural properties; hydroxyl groups promote interaction with coupling agent; good interfacial adhesion.	Lower impact energy compared to pure polymers; decreased maximum deformation at high BC content.	[35]
315	PP/BC from Wood + MAPP (3 wt.%)	Injection Moulding	Improved tensile and flexural properties (especially at 50% filler); excellent interaction with coupling agent.	Low plastic deformation; limited impact energy compared to virgin polymer.	[35]
340	PBAT/BC from Carob	Melt mixing	Good embedding in the PBAT matrix.	Reduction in tensile strength.	[32]
400	Epoxy/BC from Coffee	Mixing and Curing	-	Non-conductive behaviour.	[105]
400	Epoxy/BC from Wood Apple Shell	Mixing and Curing	Moderate abrasive wear resistance; good matrix-filler compatibility.	Formation of crack lines after wear tests (though no detachment)	[33]
400	HDPE/BC from Rice Husk	Injection Moulding	Increased impact energy.	Fragile BC particles: loss of hydroxyl groups leads to lack of interaction with MAPE.	[35]
400	PBATBC from Carob	Melt mixing	Improved filler reinforcing effect. High mechanical performance.	Lower scavenging efficiency.	[32]
400	PP/BC from Wood + MAPP (3 wt.%)	Injection Moulding	Slightly higher tensile and flexural properties compared to higher-temperature composites.	Decreased impact energy; loss of interfacial adhesion due to reduction of hydroxyl groups.	[35]
445	HDPE/BC from Wood + MAPE (3 wt.%)	Injection Moulding	Higher elastic modulus than pure polymer (though lower than BC at 315 °C).	Inferior mechanical properties compared to 315 °C treatment; poor interface; filler brittleness.	[35]
445	PP/POE + BC from Wood + MAPP (3 wt.%)	Injection Moulding	Higher elastic modulus than pure polymer (though lower than BC at 315 °C).	Inferior mechanical properties compared to 315 °C treatment; poor interface; filler brittleness.	[35]
500	HDPE/BC from Rice Husk	Injection Moulding	Highest Young’s modulus value.	-	[95]
500	PLA/BC from Hazelnut Shell + Silane coupling agent	3D Printing	Slight improvement in tensile strength.	Acts as a local heater; reduced viscosity; Newtonian behaviour with shear thinning at high frequency.	[106]
500	PP/POE + BC from Wood + MAPP (3 wt.%)	Injection Moulding	Higher complex viscosity.	No nucleation effect; reduced mechanical properties.	[100]
550	PLA/BC from Beechwood	Injection Moulding	Increased rigidity.	Reduced thermal stability; accelerated PLA degradation.	[96]
600	Epoxy/BC from Coffee	Mixing and Curing	-	Non-conductive behaviour.	[105]
600	Epoxy/BC from Wood Apple Shell	Mixing and Curing	Good abrasive wear resistance.	Formation of crack lines and particle detachment after wear tests.	[33]
600	HDPE/BC from Rice Husk	Injection Moulding	Improved thermal stability; highest tensile stress and storage modulus.	-	[95]
700	HDPE/BC from Rice Husk	Injection Moulding	Improved thermal stability; high tensile properties compared to neat HDPE.	Pore collapse; reduction in mechanical properties.	[95]
700	PE/BC from Coffee Grounds	Melt Compounding	Good compatibility: polymer confined in pores; improved thermo-oxidative stability.	Decrease in melting temperature and crystallinity degree.	[107]
700	PLA/BC from Hazelnut Shell + Silane	3D Printing	Excellent photothermal performance for the crystallisation process.	Acts as a local heater; reduced viscosity; Newtonian behaviour.	[106]
800	Epoxy/BC from Coffee	Mixing and Curing	-	Non-conductive behaviour.	[105]
800	Epoxy/BC from Wood Apple Shell	Mixing and Curing	Higher abrasive wear resistance.	Micro-void formation after wear tests (no particle detachment).	[33]
800	HDPE/BC from Rice Husk	Injection Moulding	Improved thermal stability; high tensile properties compared to neat HDPE.	Pore collapse; reduction in mechanical properties.	[95]
900	HDPE/BC from Rice Husk	Injection Moulding	Improved thermal stability.	Pore collapse; reduction in mechanical properties.	[95]
900	PLA/BC from Hazelnut Shell + Silane	3D Printing	Good photothermal performance for crystallisation.	Reduced viscosity; liquid-like Newtonian behaviour.	[106]
900	PP/POE + BC from Wood + MAPP (3 wt.%)	Injection Moulding	Higher stiffness; improved impact toughness due to compatibility; nucleation effect.	Lower complex viscosity; reduced mechanical properties.	[100]
1000	Epoxy/BC from Coffee	Mixing and Curing	Electrical conductivity (2.02 S/m); increased Young’s modulus.	Reduced elongation at break.	[105]

**Table 4:** Influence of pyrolysis temperature on the final properties of biochar-based composites, considering increasing pyrolysis temperature and different polymer matrix types.

	By Increasing Pyrolysis Temperature ↑ T_pyr_				
Property	Polyolefin	Resin	Elastomer	Polyester _fuel-based_	Polyester _bio-based_
Rheological behaviour					
Complex Viscosity	↑ [67,94]	-	↓ ^1^ [100]	↑ [32]	↓ ^2^ [106,125]
Mechanical Performance					
Young’s Modulus	↑ [107]	↓ [101]	↑ [100]	↑ [32]	↑ [106]
Tensile Strength	↑ [94,107]	↓ [101]	↓ [100]	↓ [32,104]	↓ [106,131]
Elongation at Break	↓ [107]	↑ [98,101]	↓ [100]	↑ [32]	↓ [106]
Crystallinity	↑ [95,110]	-	↑ [100]	↓ [32]	↑ [106,126]
Thermal Stability	↑ [94,95]	-	↑ [94,95,100]	↑ [32]	↓ [106]
Electrical Conductivity	↑ [115]	-	-	-	-

↑ increase; ↓ decrease; - no information available; ^1^ decrease but still higher than neat matrix; ^2^ lower than neat matrix.

## Data Availability

No new data were created or analysed in this study. Data sharing is not applicable to this article.
